# TGF-*β*1 promotes cerebral cortex radial glia-astrocyte differentiation *in vivo*

**DOI:** 10.3389/fncel.2014.00393

**Published:** 2014-11-21

**Authors:** Joice Stipursky, Daniel Francis, Rômulo Sperduto Dezonne, Ana Paula Bérgamo de Araújo, Lays Souza, Carolina A. Moraes, Flávia Carvalho Alcantara Gomes

**Affiliations:** Laboratório de Neurobiologia Celular, Programa de Biologia Celular e do Desenvolvimento, Instituto de Ciências Biomédicas, Universidade Federal do Rio de Janeiro - Centro de Ciências da SaúdeRio de Janeiro, RJ, Brazil

**Keywords:** radial glia, TGF-β, gliogenesis, neurogenesis, cerebral cortex

## Abstract

The major neural stem cell population in the developing cerebral cortex is composed of the radial glial cells, which generate glial cells and neurons. The mechanisms that modulate the maintenance of the radial glia (RG) stem cell phenotype, or its differentiation, are not yet completely understood. We previously demonstrated that the transforming growth factor-β1 (TGF-β1) promotes RG differentiation into astrocytes *in vitro* (Glia 2007; 55:1023-33) through activation of multiple canonical and non-canonical signaling pathways (Dev Neurosci 2012; 34:68-81). However, it remains unknown if TGF-β1 acts in RG-astrocyte differentiation *in vivo*. Here, we addressed the astrogliogenesis induced by TGF-β1 by using the intraventricular *in utero* injection *in vivo* approach. We show that injection of TGF-β1 in the lateral ventricles of E14,5 mice embryos resulted in RG fibers disorganization and premature gliogenesis, evidenced by appearance of GFAP positive cells in the cortical wall. These events were followed by decreased numbers of neurons in the cortical plate (CP). Together, we also described that TGF-β1 actions are region-dependent, once RG cells from dorsal region of the cerebral cortex demonstrated to be more responsive to this cytokine compared with RG from lateral cortex either *in vitro* as well as *in vivo*. Our work demonstrated that TGF-β1 is a critical cytokine that regulates RG fate decision and differentiation into astrocytes *in vitro* and *in vivo*. We also suggest that RG cells are heterogeneous population that acts as distinct targets of TGF-β1 during cerebral cortex development.

## Introduction

Radial glia (RG) cells are considered the major progenitor cell population present in the developing cerebral cortex (Kriegstein and Alvarez-Buylla, 2009).These cells have a long radial fiber that elongate from its cell body, in the ventricular zone (VZ), through the entire developing cortical wall. During the initial steps of brain development, RG cells, which are derived from the neuroepithelium, are actively proliferative cells and, by asymmetric divisions, originate neurons that migrate along their radial fibers to their specific layers at the cortical plate (CP). By the end of the neuronal migratory period, RG cells arrest their cycle and differentiate into cortical astrocytes (Munoz-Garcia and Ludwin, 1986; Voigt, 1989; Culican et al., 1990; Bentivoglio and Mazzarello, 1999; Miyata et al., 2001; Noctor et al., 2001, 2002; Götz et al., 2002; Malatesta et al., 2003; Anthony et al., 2004).

Although characteristics of RG cells such as, self-renewal and progenitor capacity, have been assured, it is widely discussed if these features can be attributed to all RG cells of the embryonic brain, or if it is restricted to specific populations of these cells (Pinto and Götz, 2007). Heterogeneity in RG cells has been described along the telencephalon regions, revealed by distinct expression of the transcription factors Pax6, Emx2 and FoxG1, which confers to these cells their neurogenic or gliogenicprogenitor property (Kriegstein and Götz, 2003; Hevner et al., 2006; Pinto and Götz, 2007). These transcription factors have been reported to be under the control of a combination of morphogen gradients along the developing axes, which determines specific telencephalon region territories (O’Leary and Sahara, 2008).

RG-astrocyte differentiation is a well-recognized event, however the mechanisms and molecules that control generation of different pools of astrocytes and neurons are still elusive. Several lines of evidence suggest that increasing neuronal pools play essential role in the control of RG maintenance and/or differentiation (Hunter and Hatten, 1995; Anton et al., 1997; Nakashima et al., 1999; Mi et al., 2001; Takizawa et al., 2001; Uemura et al., 2002; Patten et al., 2003; Schmid et al., 2003; Nishino et al., 2004; Barnabé-Heider et al., 2005; He et al., 2005; Stipursky and Gomes, 2007; Stipursky et al., 2012a). Although several soluble factors were demonstrated to control astrocytogenesis during CNS development such as leukemia inhibitor factors (LIFs) of the interleukin-6 (IL-6) family, including CNTF, LIF, and Cardiotrophin-1 (CT-1) (for revision see Stipursky et al., 2009), the role of neuronal derived soluble factors on RG-astrocyte transformation is still poorly known.

We previously reported that cerebral cortex neurons induce RG-astrocyte differentiation *in vitro* through secretion of the transforming growth factor-β1 (TGF-β1; Stipursky and Gomes, 2007; Stipursky et al., 2012a).

TGF-β1 is a multifunctional cytokine, present virtually in all tissues, that controls multiple biological and pathological events such as embryogenesis, immune response, extracellular matrix protein (ECM) production, cell differentiation and cell-cycle control in different tissues (Massagué, 1998; Massagué and Gomis, 2006). In the CNS, TGF-β1 has been reported to play key function in neuronal generation, survival and migration (Brionne et al., 2003; Miller, 2003; Espósito et al., 2005), glial differentiation(Sousa Vde et al., 2004; Romão et al., 2008), and synapse formation (Diniz et al., 2012, 2014).

TGF-β1 signaling might be mediated by the canonical pathway that involves SMADs2/3 and SMAD4 transcription factors or non-canonical signaling pathways, that involve the RasGTPAses, mitogen-activated protein kinase (MAPK), or phosphatidylinositol-3 kinase (PI3K) proteins (Javelaud and Mauviel, 2005; Massagué and Gomis, 2006). We previously reported that TGF-β1 controls RG differentiation into neurons and astrocytes by activation of SMADs/PI3K and MAPK, respectively, in distinct RG subpopulations *in vitro* (Stipursky et al., 2012a).

Although the presence of different isoforms of TGF-β molecules have already been described in the proliferative zones of the embryonic cerebral cortex (Mecha et al., 2008), there are few data regarding the expression, modulation and distribution of TGF-β receptors in RG cells *in vivo*. Further, the mechanisms that modulate neurogenesis to gliogenesis switch of RG induced by TGF-β1 are still unknown.

Here, we investigated the role of TGF-β1 on RG-astrocyte switch in the developing cerebral cortex and the implications of RG heterogeneity to this event. We showed that TGF-β1 induces premature gliogenesis and disrupts RG polarity mainly in the dorsomedial area of the cerebral cortex. For the first time, we provide evidence that specific RG subpopulations distinctly respond to TGF-β1 *in vivo*.

## Methods

### Ethical approval

All animal protocols were approved by the Animal Research Committee of the Federal University of Rio de Janeiro (DAHEICB024).

### RG cell cultures

Gestational day 14 Swiss mice embryos were collected and dissected for cerebral cortex separation. After dissection tissues were dissociated in DMEM/F12 (Invitrogen) medium and after cell counting, 105 cells were plated in 25 cm^2^ culture bottles in neurosphere growing media DMEM/F12 containing 1% glutamine, 0.1% de penicillin/streptomycin, 2% B27 (Invitrogen), 20 ng/mL EGF (Epidermal growth factor, Invitrogen) and 20 ng/mL FGFb (basic Fibroblast growth factor, R&D Systems), for 6 days, *in vitro*. The 2/3 of the media was changed every 2 days. After this period, neurospheres were enzymatically dissociated in 0.05% Trypsin/EDTA (Invitrogen), and 105 RG isolated cells were plated in glass coverslips previously coated with 50 μg/mL with poli-L-lisin (Invitrogen) and 10 μg/mL laminin (Invitrogen) in 24 wells culture plates. Cells were kept in DMEM/F12 containing 1% glutamine, 0.1% penicillin/streptomycin, 2% de B27 (Invitrogen), 20 ng/mL EGF (Invitrogen) and 20 ng/mL FGFb (R&D Systems) for 24 h. After this period, cells were treated with 10 ng/mL of TGF-β1 (R&D Systems) or 10 μm of SB431542 (Sigma Aldrich) in medium, without mitogenic factors, for 24 h.

### *In utero* intraventricular injection

*In utero* intraventricular injections of E14 mice embryos were performed as described by Walantus et al. (2007). Pregnant Swiss mice in the 14 gestational day were anesthetized with intraperitoneal injection of 2-2-2 Tribromoethanol (Sigma Aldrich) 1 mg/g of body weight. After anesthesia, females were subjected to surgical procedure, in which the uterus was exposed. After visualization of the embryos, they were manually positioned to allow observation of brain hemispheres. Each embryo was subjected to intraventricular injection inside the lateral ventricles of 2 μl of control solution (PBS, 0.05% BSA, 0.025% Fast Green [Sigma Aldrich]), or solution containing 100 ng of TGF-β1 (R&D Systems) or 10 μM of SB431542 (Sigma Aldrich), using glass micropipettes. After injections, the uterus was repositioned inside abdominal cavity and abdominal muscle and skin layers sutured. Bromodeoxiridine (BrdU, Sigma Aldrich) was intraperitoneally injected in the preagnant mouse after 2 and 24 h of surgery, to follow cells generated from RG just after TGF-β1 stimulation and to analyze long lasting effects in RG population. Forty-eight hours after surgery, the female was sacrificed and embryos were perfused with ice cold 4% paraformaldehyde (PFA). Brains were collected and processed for immunohistochemistry and real time RT-PCR.

### Immunocytochemistry and immunohistochemistry

After culture, cells were fixed in 4% PFA (Vetec) for 15 min. After this period, cells were extensively washed in PBS (phosphate buffered saline) and permeabilized with 0.2% Triton X-100 (Vetec) for 5 min at room temperature. Cells were then incubated with blocking buffer containing 3% serum bovine albumin (BSA), 5% normal goat serum (NGS) (Sigma Aldrich) diluted in PBS for 1 h, followed by 12 h incubation with primary antibodies at 4°C diluted in the same solution. After this period, cells were extensively washed in PBS and incubated with secondary antibodies for 2 h at room temperature. Nuclei were labeled with DAPI (4’, 6-Diamidino-2-phenylindole; Sigma Aldrich), or Draq5 (Pierce). Glass coverslips were mounted in glass slides with Faramount mounting media (DakoCytomation), and stained cells were visualized using a fluorescent optical microscope Nikon TE3000. For immunohistochemistry, brain were fixed in 4% PFA for 48 h, and subjected to vibratome sectioning, to obtain 40 μm sections. After sectioning, floating brain slices were incubated with blocking buffer for 1 h under shaking. After incubation with primary antibodies for 12 h at 4°C, followed by extensive washing in PBS, slices were incubated with secondary antibodies for 2 h at room temperature under shaking. Primary antibodies were: mouse anti-Nestin (Chemicon, 1:100), rabbit anti-BLBP (Chemicon, 1:200), rabbit anti-ErbB2 (Santa Cruz Biotechnology, 1:200), rabbit anti-Notch1 (Cell Signaling, 1:500), rabbi anti-TGFRII (Santa Cruz Biotechnology, 1:100), rabbit anti- phophoSmad 2/3 (Santa Cruz Biotechnology, 1:50), rabbit anti-Laminin (Sigma Aldrich, 1:100), rabbit anti-GFAP (Dakocytomation, 1:500), mouse anti-β TubulinIII (Promega, 1:1,000), rabbit anti-Doublecortin (Abcam, 1:200), rati anti-BrdU (Accurate, 1:1,000), rabbit anti-Foxg1 (Santa Cruz Biotechnology, 1:200). Secondary antibodies were conjugated to AlexaFluor 488, AlexaFluor 546, and AlexaFluor 633 (Invitrogen Molecular Probes). Nissl Trace Green (Molecular Probes, 1:1,000) staining was used to label neuronal cell soma. Images of labeled tissue were obtained using a Leica SP5confocal microscope.

### Western blot

Protein levels were analyzed as previously described (Dezonne et al., 2013). After dissection, cerebral cortex tissues from Swiss mice embryos were lysed in RIPA buffer [20 mMTris-HCl (pH 7.5); 150 mMNaCl; 1 mM Na/EDTA; 1 mM EGTA; 1% NP-40; 1% sodium deoxycholate; 2.5 mM sodium pyrophosphate; 1 mMglycerophosphate; 1 mM Na_3_VO_4_; 1 μg/mLleupeptin]. Cell suspension was homogenized, sonicated, and centrifuged for 10 min at 14,000 rpm in a refrigerated centrifuge. Subsequently, the supernatant was collected and the protein dosage was performed using the BCA Protein Assay kit (Pierce, Rockford, Ill., USA). A total of 20 μg of protein was loaded per lane and submitted to electrophoretic separation in a 10% SDS-PAGE gel. After separation, proteins were electrically transferred onto a nitrocellulose transfer membrane (Protran, Dassel, Germany) for 1 h. The membrane was blocked in 5% BSA in Tris-buffered Tween 20 (TBS-T; Merck, Darmstadt, Germany) and primary antibody incubation was performed overnight at 4°C followed by peroxidase-conjugated secondary antibody incubation for 1 h at room temperature. Proteins were visualized using the enhancing chemiluminescence detection system (SuperSignal West Pico Chemiluminescent Substrate; Thermo Scientific, Rockford, Ill., USA) and nitrocellulose membranes were exposed to autoradiographic films (Kodak, São José dos Campos, Brazil). Primary antibodies were: mouse phosphoSmad2 (Cell Signaling; 1:1,000), rabbit anti-ErbB2 (Santa Cruz Biotechnology 1:200); rabbit anti-TGFRII (Santa Cruz Biotechnology; 1:200); mouse anti-α-tubulin (Sigma Aldrich; 1: 5,000). The secondary peroxidase-conjugated antibodies were: goat anti-rabbit IgG and goat anti-mouse IgG (Amersham Biosciences, Piscataway, N.J., USA; 1: 3,000). After protein detection, densitometric analysis of autoradiographic films was done using Image J 1.48 software. Each experiment was done in triplicate, and proteins were loaded in triplicate in SDS-PAGE gel.

### Real time RT-PCR

Total RNA was isolated from embryonic mice cerebral cortex using Direct-zol™ RNA MiniPrep (ZymoReserch, USA) according to the protocol provided by the manufacturer, and quantified using NanoDrop ND-1000 Spectrophotometer ThermoFisherScientific, USA).Two micrograms of total RNA were reverse transcribed with RevertAid first Strand cDNA Synthesis Kit according to the manufacturer (Thermo Fisher Scientific, USA). Sense and antisense specific for FoxG1, and β-actin genes were used. β actin sense: TGG ATC GGT TCC ATC CTG G, anti-sense: GCA GCT CAG TAA CAG TCC GCC TAG A; FoxG1 sense: CGA CAA GAA GAA CGG CAA GTA CGA, anti-sense: AGC ACT TGT TGA GGG ACA GGT TGT. Sequences were verified to be specific using Gen Bank’s BLAST (Altschul et al., 1997). Quantitative real-time RT-PCR was performed using Maxima SYBR green qPCR Master Mix (Thermo Scientific, USA). Reactions were per formed on ABI PRISM 7500 Real Time PCR System (Applied Biosystems). The relative expression levels of genes were calculated using the 2^−ΔΔCT^ method (Livak and Schmittgen, 2001). The amount of target genes expressed in a sample was normalized to the average of thee ndogenous control.

### Statistical analysis

Statistical analyses were done using one-way non-parametric ANOVA coupled with Tukey post-test by GraphPad Prism 4.0 software, and *P* < 0.05 was considered statistically significant. The experiments were performed in triplicate, and each result represents the mean of at least 4–6 animals analyzed.

## Results

### RG cells are potential targets of TGF-β1 *in vivo*

**Figure 1:**
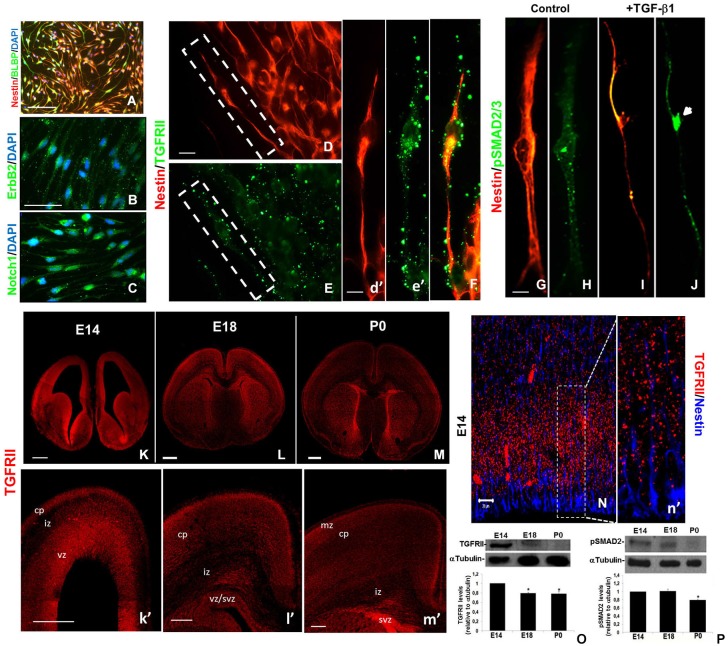
**RG cells express the TGF-β1 signaling pathway members**. RG enriched cell cultures were isolated from cerebral cortex, grown into neurospheres and analyzed for specific cellular markers. Under these conditions the cells exhibit the typical RG cell elongated morphology and staining for specific markers: nestin/BLBP **(A)**; ErbB2 **(B)**; and Notch1 **(C)**. RG also expresses TGFRII in their membranes **(D–F)**. Treatment of these cells with 10 ng/mL of TGF-β1 induces phosphorylation and nuclear translocation of Smad2/3 **(G–J)**. Note that TGFRII is expressed predominantly in VZ/CP (E14),VZ/SVZ/CP (E18), and SVZ/CP (P0) in mice **(K–M, k’–m’)**. TGFRII is distributed as a punctate pattern all over nestin+ RG cell bodies and fibers **(N, n’)**. Western blotting assays revealed that either TGFRII **(O)** as phosphorylated Smad2 **(P)** is down regulated during cerebral cortex development. vz:ventricular zone, svz: subventricular zone, cp:cortical plate. *P < 0.05. Scales: 50 μm **(A)**, 20 μm **(B)**, 10 μm **(D, I)** 500 μm **(K–M)**; 200 μm **(k’–m”)** 20 μm **(N)**.

In order to investigate RG cells responsiveness to TGF-β1, we first identified the TGF-β receptor type II (TGFRII) in RG cells *in vitro* and *in vivo*. To do that, we performed RG isolation from neurospheres derived E14 mice embryos cerebral cortex. Under this culture condition, these cells present a typical radial morphology and label for Nestin, BLPB, Notch1 and ErbB2 (Figures 1A–C), attesting their RG cells phenotype. We also detected high staining for TGFRII in their membranes (Figures 1D–F). Treatment of RG culture with TGF-β1 induced phosphorylation and nuclear translocation of Smads2/3, a hallmark of TGF-β1 signaling pathway activation (Figures 1G–J).

Immunohistochemical assays of the mouse brain revealed that TGFRII is more robustly expressed in the VZ (ventricular zone) and CP (cortical plate) of E14 and in the same layers as well as in SVZ (subventricular zone) of E18 and P0 mice cerebral cortex (Figures 1K–M,k′–m′). We identified a punctate TGFRII staining in RG cell body and processes in the E14 telencephalon (Figures 1N,n′). Western blotting analysis revealed that TGFRII is negatively modulated during development, since this protein is present at high levels in E14 telencephalon, is slightly detectable in E18 and tend to disappear in P0 (Figure 1O). The down regulation of TGFRII overlaps with the amount of phospho Smad2 at P0 (Figure 1P). Together, this data suggest that RG cells might be target of TGF-β1 actions *in vitro*, as well as *in vivo*.

### Intraventricular injection of TGF-β1 disrupts polarity of RG cells

RG cell elongated morphology is a critic characteristic that allows neuronal migration and correct positioning in the CP within the different layers of the cerebral cortex (Rakic, 1971, 1995; Hatten, 1999; Yokota et al., 2007; Radakovits et al., 2009). Loss of this typical morphology is a hallmark of RG-astrocyte differentiation.

**Figure 2:**
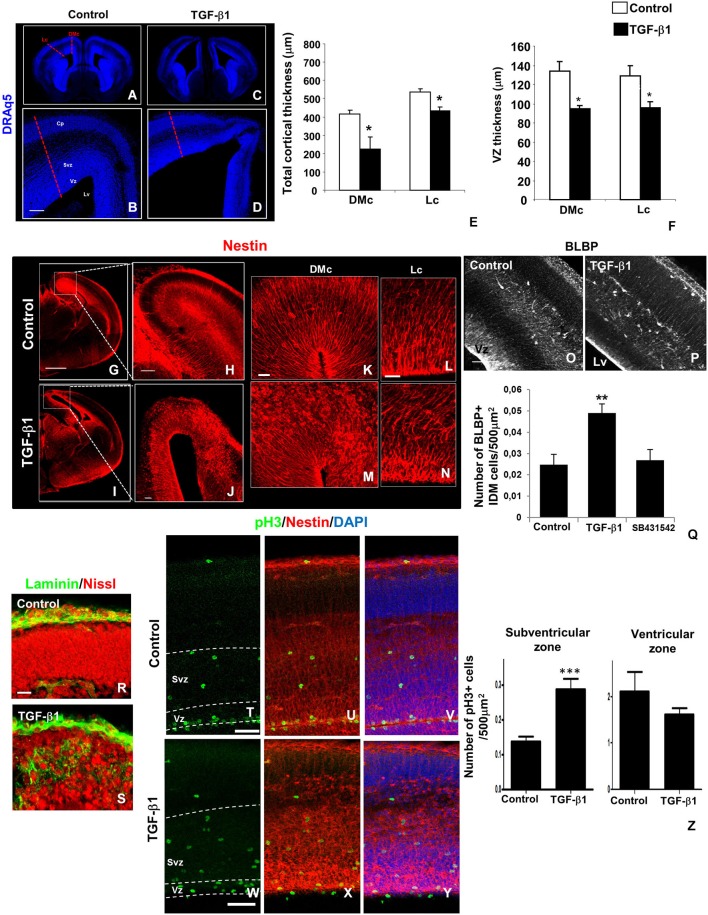
**TGF-β1 injection affects cerebral cortex and RG morphology**. *In utero* intraventricular injection of TGF-β1 in mouse embryos (injection at E14 and analysis at E16) promoted several morphological alterations in the cerebral cortex wall in lateral (Lc) and dorsomedial (DMc) cortex areas **(A–D)**. TGF-β1 reduces the thickness of total cortical area **(E)** and VZ **(F)** in both regions. Note that TGF-β1 also disrupted nestin+ (red) radial fiber networks **(G–J)**, an event more prominent at DMc than in Lc **(K–N)**. RG loss of polarity induced by TGF-β1 is accompanied by increase in the numbers of BLBP+ cells (white) with intermediate differentiated morphology (IDM) across cortical wall **(O–Q)**. SB431542 injection does not affect BLBP+ IDM cells numbers. These morphological alterations were followed by basal membrane laminin (green) and neuronal cell bodies (Nissl) ectopic distribution **(R,S)**. TGF-β1 also disorganized pH3+ cells (green) distribution across cortical wall, especially VZ pH3+ cells’ nucleus alignment **(T–Y)**, without affects its numbers **(Z)**, however increased the numbers of pH3+ cells in the SVZ **(Z)**. *P < 0.05, **P < 0,005, ***P < 0005. Scales: 500 μm **(G)**, 100 μm **(B,O)**, 50 μm **(H,J,T,W)**, 20 μm **(K,L,R)**. Cp: cortical plate, Vz: ventricular zone, Svz: subventricular zone, Cx: cortex, Lv: lateral ventricle.

Intraventricular injection of TGF-β1 resulted in profound morphological alterations especially in the telencephalon, resulting in dilated lateral ventricles, and evident reduction of cortical wall thickness in dorsomedial (DMc) and lateral (Lc) areas of the cortex (Figures 2A–E).We also observed reduced VZ thickness in TGF-β-injected brains compared with vehicle solution injected brains (Figure 2F).These thickness reduction is observed along rostral to caudal regions of the cerebral cortex (data not shown).Interestingly, these morphological alterations were accompanied by severe disorganization of nestin labeled-RG fibers in TGF-β1-injected brains (Figures 2G,J). This disorganization characterized loss of polarity of the radial processes and was more prominently observed in DMc rather than in Lc areas of the cortex (Figures 2K–N).

In addition to RG fibers displacement, TGF-β1 also promoted an increment in approximately 98% on BLBP-labeled cells witha morphology similar to glial progenitors, in the midway of their differentiation path, which we called RG intermediate differentiation morphology (IDM; Figures 2O–Q). Injection with pharmacological inhibitor of TGF-β1 signaling pathway SB431542 did not affect BLBP+ IDM cells generation (Figure 2Q). We also observed that TGF-β1 caused ectopic laminin distribution in the pial region of the cortical wall (Figures 2R,S).These phenotypes were also associated with increasing numbers of pH3+ cells in SVZ, but not in VZ (Figures 2T–Z). In addition RG fibers disorganization were also followed by displacement of pH3+ cells at VZ, leading to ectopic positioning of these proliferative cell’s nucleus (Figures 2T,W).

These data shows that TGF-β1 regulates cerebral cortex thickness, RG morphology and polarity and progenitor positioning, and suggest that these events might be associated to regulation of basal lamina structure, an issue clearly related to RG cell polarity.

### TGF-β1 promotes premature gliogenesis in dorsomedial (DMc) area of the cerebral cortex

**Figure 3:**
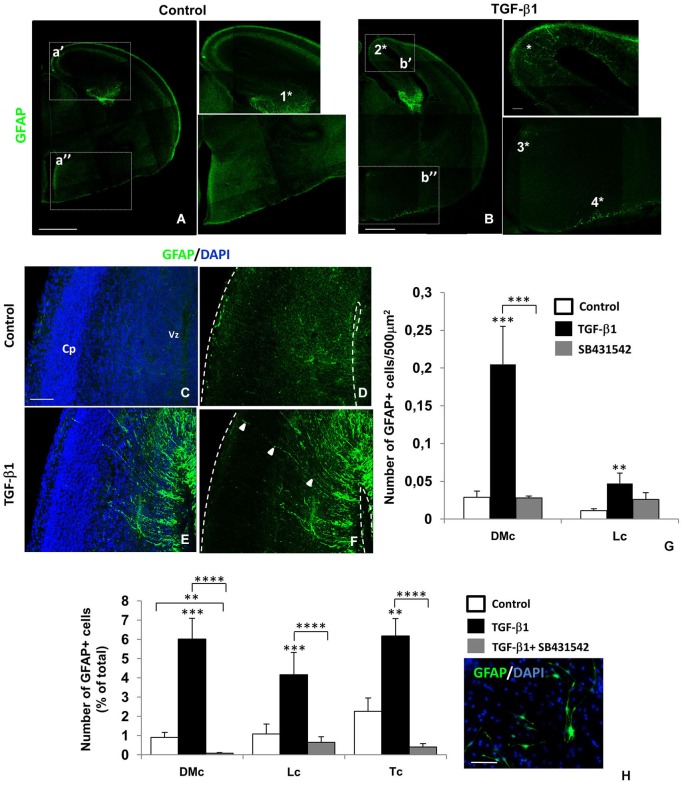
**TGF-β1 promotes premature gliogenesis in the cerebral cortex**. Intraventricular injection of TGF-β1 in mouse embryos (injection at E14 and analysis at E16) caused premature appearance of GFAP+ cells (green) in different telencephalon regions: dorsomedial cortex/cingulate cortex (2*), neuroepithelium related to the third ventricule (3*) and pial surface of the preoptic area (4*). At the hippocampal formation (1*), GFAP labeling was not affected. TGF-β1 induced gliogenesis was more evident at the dorsomedial area of the cerebral cortex (DMc), than in lateral cortex (Lc) **(C–G)**. Note the GFAP+ (green) radial fibers of differentiating cells (arrows, **F**). In radial glia (RG) isolated cultures, TGF-β1 also promoted appearance of GFAP+ cells in a greater extend in DMc than in Lc and total cortex (Tc) **(H)**. ***P < 0.0005, *P < 0.005. Scales: 500 μm **(A,B)**, 50 μm **(C,H)**. Cp: cortical plate, Vz: ventricular zone.

We previously demonstrated that TGF-β1 controls RG differentiation into astrocytes and neurons by distinct signaling pathways *in vitro* (Stipursky et al., 2012a). In order to assess the fate of RG under the influence of TGF-β1 *in vivo*, we took the advantage of *in utero* intraventricular injection technique. Injection of TGF-β1 inside the lateral ventricles of mouse embryos also caused robust premature astrocyte generation (Figure 3). In the telencephalon TGF-β1 injection caused appearance of GFAP+ cells in distinct regions compared with vehicle injected brains (Figures 3A,B), such as the cingulate cortex (2*) neuroepithelium related to the third ventricle associated with the ventral diencephalic sulcus (3*), and also at the pial region of the preoptic area (4*).In the evident hippocampal neuroepithelium there was no difference in GFAP labeling pattern in control and TGF-β1 injected brains (1*).

Apart from other regions, we observed that in DMc area of the cerebral cortex (cingulate cortex) astrocytogenesis was more evident. The appearance of GFAP+ cells bearing a yet radial-like morphology in this area (Figures 3C–F) suggest that TGF-β1 induced RG cells to adopt an astrocyte phenotype.

Astrocyte differentiation was significantly increased by TGF-β1 in the DMc area in comparison to the lateral area of the cerebral cortex (15 X; Figure 3G). Injection of a pharmacological inhibitor of TGF-β receptor, SB431542, did not affect the gliogenesis in this area (Figure 3G).

In order to confirm the specificity of TGF-β1 actions in different cortical areas, we generated cultures of isolated RG cells from DMc and Lc areas and from total cortex (Tc). We observed that DMc cells were more responsive to TGF-β1 astrocytogenic induction, than Lc cells. The number of GFAP+ cells increased by 5 times in DMc cells treated with TGF-β1 whereas only 3 times in Lc cells. For Tc cells, the increasing in GFAP+ cell numbers was compared to those found in DMc-treated condition (Figure 3H).

Thus, RG from different cerebral cortexareas respond to TGF-β1 by acquiring the astrocytic phenotype.

### TGF-β1 affects neurogenesis and neuronal positioning in cortical plate

Neurogenesis and neuronal migration are events that occur during specific time window in the developing cerebral cortex; both events directly dependent of RG cell stem cell and scaffold properties, respectively (Rakic, 1971; Costa et al., 2010; Vogel et al., 2010; Sild and Ruthazer, 2011; Stipursky et al., 2012b). We previously described that as well as astrocytogenesis, neurogenesis can be controlled by TGF-β1 by activation of canonical and non-canonical signaling pathways, respectively (Stipursky et al., 2012a). Although neurogenesis was reported to involve TGF-β1 action *in vitro* (Vogel et al., 2010), it is not known if this factor controls RG neurogenic potential *in vivo*. In order to address this question, we have performed intraventricular injection of TGF-β1.

**Figure 4:**
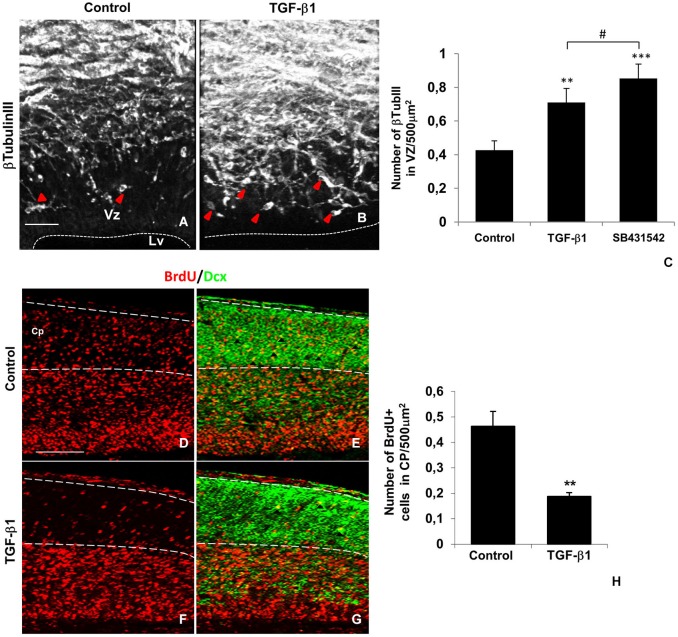
**TGF-β1 affects neurogenesis and neuronal positioning in the lateral cortex**. TGF-β1 injections (injection at E14 and analysis at E16) increased neurogenesis at the lateral cortex Vz, as shown by the presence of βTubulinIII+ cells (white, arrows) in this layer **(A–C)**. TGF-β1 decreased the number of BrdU+ cells (red) at the cortical plate (Cp) of the lateral cortex **(D,F,H)**. Note that this layer is enriched in Doublecortin+ neurons (green) that were generated at Vz and migrated to the Cp **(E,G)**. *P < 0.05, #*P* = 0.063. Scales: 50 μm **(A,D)**. Vz: ventricularzone, Lv: lateral ventricle.

TGF-β1 also affected neuronal generation and placement in CP of the Lc. Interestingly, numerous βTubulinIII+ cells were present in the VZ of TGF-β1-injected brains, counting for an 66% increment (Figures 4A–C), thus suggesting enhanced neurogenesis in this RG cell bodies enriched layer. Pharmacological inhibition of TGF-β1 signaling pathway by SB431542 injection yielded a greater enhancement of βTubulinIII+ cells numbers in VZ, compared to control condition. In order to access if this increment was due to generation of new neurons, we have labeled the cells for BrdU and Doublecortin, which label recent generated neurons from RG cells that migrated through cortical wall and reached their final destination in the CP (Pramparo et al., 2010). We observed a 55% decrease in the number of BrdU+cells in the Lc CP of TGF-β1 injected brains (Figures 4D–H), thus demonstrating that both neuronal migration and positioning are modulated by TGF-β1 *in vivo*.

### TGF-β1 controls the expression of FoxG1 in different cortical areas

Differences between the distinct regions of the brain are mainly generated during developmental controlled axis patterning-related morphogen distribution. Cerebral cortex arealizationor patterning is controlled by the expression of a great repertoire of transcription factors that define neural stem cells and progenitors generation, self-renewal and phenotypes. Those factors, such as FoxG1, are modulated by diverse morphogenetic proteins distinctly distributed in different patterning centers (Takahashi and Liu, 2006; O’Leary and Sahara, 2008).

**Figure 5:**
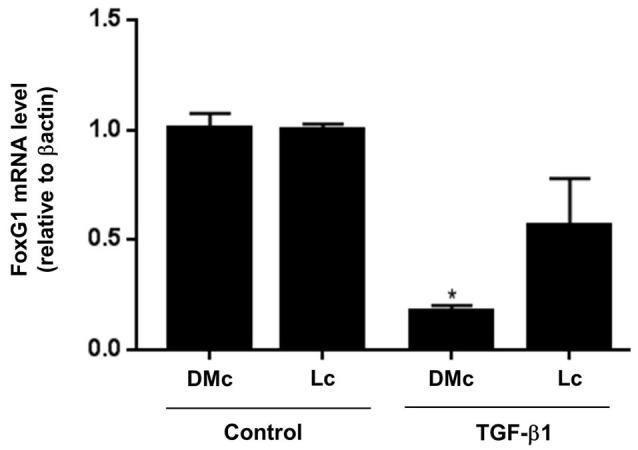
**TGF-β1 controls FoxG1 expression in the cerebral cortex**. Cerebral cortex tissue from TGF-β1 injected brains (injection at E14 and analysis at E16) were dissected to separate DMc and Lc areas and evaluated for FoxG1 mRNA expression by real time RT-PCR. TGF-β1 injections greatly decreased FoxG1 gene mRNA levels only in DMc area compared to control brains. *P < 0.01.

Quantitative analyses by real time RT-PCR of DMc and Lc tissues revealed that TGF-β1 distinctly modulated the levels of FoxG1 mRNA transcription factors in these regions. Whereas TGF-β1 reduced the expression level of FoxG1 in DMc by 80%, it had no effect in Lc (Figure 5). These results suggest that TGF-β1 controls the expression of a transcription factor related to cortical arealization *in vivo*.

## Discussion

In this study, we provide evidence for the role of TGF-β1 as a modulator of RG-astrocyte differentiation *in vivo*. Our data is pioneer in two aspects: (1) by demonstration of TGF-β1 action in radial-glial-astrocyte differentiation *in vivo*; (2) by showing distinct effects of TGF-β1 in different subpopulations of RG cells. First, we demonstrated that RG cells express the TGF-β receptor and activate the Smad pathway in response do TGF-β1. Then, we demonstrated that TGF-β1 disrupts RG cells polarized morphology and promotes premature astrocytogenesis and neuronal displacement in specific areas of the cerebral cortex. Our findings show that RG cells are potential targets for TGF-β signaling pathway and suggest that these effects are region dependent. Our data not only contribute to the understanding of the mechanism underlying fate decision and specific phenotype acquisition in the cerebral cortex, but support the hypothesis of the existence of distinct RG subpopulations with different potentials in the cerebral cortex.

### RG cells as potential targets of TGF-β1 *in vivo*: impact on RG polarity and astrocytic differentiation

Evidence suggests that VZ cells are direct targets of different TGF-β family members (Miller, 2003; Mecha et al., 2008), however, the cellular pattern of expression of TGF-β1 signaling pathway members in the developing CNS has not been well characterized. Here, we have shown TGFRII expression in the developing telencephalon, specifically in the VZ/SVZ of the cerebral cortex. Additionally, we precisely identified its distribution in RG soma and fibers, an issue only previously suggested by other authors (Miller, 2003). Moreover, the levels of TGFRII and one of its downstream effectors, phosphorylated Smad2, seems to be negatively modulated through development. These results are corroborated by previous data that showed TGF-β1 and Smad2/3 proteins expression in different CNS regions including cerebral cortex VZ, neurons and progenitor layers *in vivo* (Miller, 2003; Sousa Vde et al., 2004; Mecha et al., 2008; Powrozek and Miller, 2009). In addition, our data is in accordance with previous reports that demonstrated that TGF-β signaling members are expressed in higher levels in early moments of the telencephalon development, and that are determining for the generation of different cell types of the CNS and other regions (Luukko et al., 2001).

RG cell polarity and radial processes extension are essential characteristics that are directly related to RG maintenance of its progenitor potential and scaffold property for neuronal migration (Rakic, 1971). RG differentiation into astrocytes involves disruption of its polarity and gradual acquisition of immature astrocyte morphology (Voigt, 1989; Hartfuss et al., 2001). Here, we have shown that TGF-β1 induces specific disorganization of nestin positive RG fibers and displacement of their cell nucleus labeled for pH3. Moreover, we observed the appearance of BLBP positive cells bearing an intermediate morphology between RG and astrocytes throughout the cortical wall.

Several mechanisms have been proposed to control RG cell polarity and correct positioning of migrating neurons such as modulation of cytoskeleton molecules (Yokota et al., 2007, 2009, 2010; Weimer et al., 2009) and ECM signal transduction (Haubst et al., 2006; Voss et al., 2008). Here we observed that disruption of RG polarity induced by TGF-β1 is followed by impaired organization of the basal membrane that covers pial surface of the telencephalon, where RG cells attach their pial process endfeet (Götz and Huttner, 2005). Laminin labeling revealed an ectopic distribution pattern of this protein in pial region of the cerebral cortex, associated with deficiencies in CP formation and displaced cell bodies. Our data is supported by previous results that TGF-β1 is a potent regulator of the synthesis of laminin, fibronectin, the adhesion protein nCAM and integrins (Brionne et al., 2003; Siegenthaler and Miller, 2004; Gomes et al., 2005). Further, similar phenotypes were found in mutant mice for C3G protein, a guanine nucleotide exchange factor for small GTPases of the Ras family, and also in laminin γ1III4 mutant (Haubst et al., 2006; Voss et al., 2008). In these mice, it is observed a robust loss of radial cell polarity, disruption of basal membrane and neuronal migration and CP deficits. Thus, although we cannot fully rule out additional mechanisms, our data strongly suggested an association between TGF-β1-control of laminin organization and maintenance of RG polarity.

### TGF-β1 promotes premature gliogenesis in dorsomedial area of the cerebral cortex: implications for RG heterogeneity

In rodents, by the end of gestation, RG-astrocyte differentiation, is characterized within several molecular mechanisms by replacement of RG markers, such as BLBP and nestin, by astrocytic markers such as GFAP, the glutamate transporter GLAST and the calcium binding protein S100β (Dahl, 1981; Pixley and de Vellis, 1984).The correct timing of RG-astrocyte transformation is a crucial step to ensure correct number of neurons and cerebral cortex lamination. Here, we report that activation of TGF-β1 pathwayled to a premature appearance of GFAP+ cells in different regions of the embryonic telencephalon, mainly, in the cingulate cortex, neuroepithelium related to the third ventricle, and also at the pial region of the preoptic area. Although it has been reported the expression of TGF-β isoforms and also its different roles in these regions (Bouret et al., 2004; Dobolyi and Palkovits, 2008; Srivastava et al., 2014), the role of TGF-β1 in dorsomedial area of the cerebral cortex, cingulate cortex, specifically on astrocyte differentiation, is poorly known.

Here the reported event was region-dependent since in DMc area the appearance of GFAP+ cells and disruption of RG processes were more robust than in Lc area. This observation might be related to 2 alternatives: (1) distinct responsiveness of different brain regions to TGF-β1; (2) heterogeneity of radial glial cells. The first possibility is supported by our previous report that GFAP gene promoter from different brain regions distinctly responds to TGF-β1 (Sousa Vde et al., 2004). It is also possible that TGF-β1 might exert its actions controlling size of a brain area (Falk et al., 2008) by acting into the different subpopulations of RG cells and other progenitors previously described to contribute to cell diversity in CNS (Pinto and Götz, 2007; Stancik et al., 2010), and that this event accounts for diversity in the responsiveness to TGF-β1. Whether this is due to different levels of TGF-β receptor or intracellular signaling molecules, or even, by cell autonomous defined potentials, remains to be determined.

Several molecules have been described to guarantee the maintenance of RG self-renewal, BLBP expression and morphology characteristics, such as the proteins of Neuregulin family and its receptor ErbBs, and Notch1 (Gaiano and Fishell, 2002; Patten et al., 2003; Schmid et al., 2003; Yoon et al., 2004; Anthony et al., 2005; Ghashghaei et al., 2006, 2007). Thus, alterations of ErB2 and Notch1 expression in RG cells could lead to a premature astrocyte differentiation under TGF-β1 influence. This hypothesis is supported by reports that interaction between TGF-β1 signaling pathway proteins and radializing factors such as Notch intracellular cleaved domain (NICD) and ErbB4 is necessary to regulate the expression of target genes in neural precursors (Blokzijl et al., 2003) and the correct time of gliogenesis (Sardi et al., 2006). The exact mechanisms by which TGF-β1 pathway controls RG-astrocyte differentiation in the dorsomedial area of the cerebral cortex will require further investigation.

We reported here that activation of TGF-β1 signaling pathway in the cerebral cortex down regulates the expression of FoxG1 in DMcarea.FoxG1 is a member of the forkhead family of transcription factors, expressed by cells with high proliferation rates; it controls neurogenesis, by maintaining the undifferentiated state of neural progenitors (Dou et al., 2000; Siegenthaler and Miller, 2008). In addition, FoxG1 is mainly expressed in lateral areas of the mice cerebral cortex (Miller, 2003).Mutant mice models for FoxG1 functions share several similarities with many of the phenotypes described here, including reduction of cortical thickness and layers of the dorsal area. For example, mutant mice for FoxG1, present reduction of dorsal area, and pronounced increase of BMPs, a member of TGF-β family, expression in the telencephalon (Takahashi and Liu, 2006). Further, FoxG1 was described as a potent inhibitor of TGF-β signaling due to its association with Smad proteins (Dou et al., 2000; Siegenthaler and Miller, 2008). Although TGF-β1 affects more robustly DMc area, we also observe the effect of this factor in Lc, such as mild RG fibers morphology and neurogenesis induction, it is possible that other transcription factors responsible for arealization of the cortex might mediate TGF-β1 actions in Lc (O’Leary and Sahara, 2008).

Thus, it is possible that TGF-β1 controls the balance between gliogenesis and neurogenesis by modulating the expression and activation of different transcription factors *in vivo*. Since FoxG1 is a lateral transcription factor, a gliogenic inhibitor, and negatively regulates Smads signaling, it is possible that FoxG1 is a mediator of TGF-β1 signaling in DMc.

Besides the role of TGF-β1 in the modulation of transcription factors at transcriptional level, it is possible that the lateral morphogen gradients might exert an inhibitory action on medial ones. It correlates with our observation that endogenous TGF-β signaling pathway might not be active or engaged in promotion of astrocytogenesis at this developmental stage, since pharmacological inhibition of endogenous TGF-β signaling by SB431542 did not affect RG morphological phenotype, as well as GFAP + cells numbers. Although we have shown that TGF-β1 is a potent inductor of astrocyte differentiation (Stipursky and Gomes, 2007; Stipursky et al., 2012a), this data confirm that RG cells are mainly committed in promoting neurogenesis at this stage (Noctor et al., 2001).

### TGF-β1 affects neurogenesis and neuronal positioning in the cortical plate

Injection of TGF-β1 decreased the number of BrdU+ cells in the developing CP of the lateral area of the cortex. This effect might be the consequence of neurogenesis and/or migration deficits. The last hypothesis is more likely, since increased number of βTubulinIII+ cells was observed in the VZ, and although in the present work we cannot completely guarantee the identity of the pH3+ cells in the SVZ, it is possible that these cells could also contribute to neurogenic effect promoted by TGF-β1.

The role of TGF-β1 in neurogenesis is controversial; whereas it has been shown as inductor of neurogenesis in the cerebral cortex during embryonic stage and in the adult hippocampus (Vogel et al., 2010; Stipursky et al., 2012a; He et al., 2014); others have reported its action as negative modulator of neurogenesis in the adult SVZ (Roussa et al., 2004; Wachs et al., 2006; Siegenthaler and Miller, 2008). Although TGF-β1 has been shown to induce radial neuronal migration in the cerebral cortex, its effect in RG cell has not been previously addressed (Siegenthaler and Miller, 2004). Here we suggest that although TGF-β1 promotes neuronal generation from RG cells and as we previously demonstrated *in vitro* (Stipursky et al., 2012a), the morphological alterations triggered in radial processes in the lateral area of the cortex, even in a less extension that in DMc area, counteracts its effect and prevent neuronal migration and the accuracy in the establishment of these new generated neurons in the CP.

It is interesting that pharmacological inhibition of TGF-β1 signaling pathway by injection of SB431542 yielded an even greater increase of βTubulinIII+ cells in VZ, when compared with TGF-β1 injected brains. Although apparently contradictory, this result might indicate that endogenous TGF-β signaling pathway might be committed to control neuron generation in cerebral cortex during the neurogenic stage of the CNS development (Vogel et al., 2010). Further, it is possible that different levels of TGF-β signaling activation might be critical to elicit positive or negative responses to this factor. Accordingly, it has been demonstrated that opposite actions of TGF-β1 in neuronal migration is concentration dependent (Siegenthaler and Miller, 2004).

Together our results points to a new feature of TGF-β1 action in patterning the developing telencephalon. By acting in different RG populations, TGF-β1 promotes the generation of astrocytes and/or neurons in a regional dependent manner. Deficits in pathways that operate in RG physiology might generate dysfunctional cells, disorders in neuronal migration and premature astrocytogenesis, leading to diverse types of lamination defects in the developing cortex, such as observed in Lisencephaly and the congenital abnormality cortical dysplasia. Identification and characterization of the mechanisms underlying RG maintenance and differentiation might contribute to generation of therapeutic approaches to cell restocking in CNS parenchyma.

## Conflict of interest statement

The authors declare that the research was conducted in the absence of any commercial or financial relationships that could be construed as a potential conflict of interest.

## Abbreviations

BLBP: brain lipid binding protein
BMP: bone morphogenetic protein
BrdU: bromodeoxiuridine
BSA: bovine serum albumin
CNS: central nervous system
CP: cortical plate
DAPI: 4_,6-diamidino-2-phenylindole dihydrochloride
DMc: dorsomedial cortex
DMEM/F12: Dulbecco’s modified Eagle’s medium supplemented with nutrient mixture F-12
E14: embryonic day fourteen
E18: embryonic day eighteen
P0: postnatal day 0
ECM: extracellular matrix
EGF: epidermal growth factor
FGFb: basic fibroblast growth factor
GFAP: glial fibrillary acidic protein
IDM: intermediate differentiating morphology
Lc: lateral cortex
LV: lateral ventricle
MAPK: mitogen-activated protein kinase
PBS: phosphate-bufferedsaline
PI3K: phosphatidylinositol-3 kinase
RG: radial glia
SMAD: homologue protein for SMA protein from *C. elegans* and mothers against decapentaplegic (MAD) from *Drosophila*
SVZ: subventricular zone
TBS-T: Tris-bufferedsaline- Tween20
Tc: total cortex
TGFRII: transforming growth factor beta type II receptor
TGF-β1: transforming growth factor beta 1
VZ: ventricular zone.
