# Corrigendum: TGF-β1 promotes cerebral cortex radial glia-astrocyte differentiation *in vivo*

**DOI:** 10.3389/fncel.2015.00232

**Published:** 2015-06-18

**Authors:** Joice Stipursky, Daniel Francis, Rômulo S. Dezonne, Ana P. B. de Araújo, Lays Souza, Carolina A. Moraes, Flávia C. A. Gomes

**Affiliations:** Programa de Biologia Celular e do Desenvolvimento, Laboratório de Neurobiologia Celular, Instituto de Ciências Biomédicas, Universidade Federal do Rio de Janeiro - Centro de Ciências da SaúdeRio de Janeiro, Brazil

**Keywords:** radial glia, TGF-β, gliogenesis, neurogenesis, cerebral cortex

The last author Flavia Carvalho Alcantara Gomes appears with the incorrect citation name in this article (Stipursky et al., 2014). The correct citation name for this author is Gomes FC.

## Funding

Fundação Carlos Chagas Filho de Amparo à Pesquisa do Estado do Rio de Janeiro (FAPERJ), Conselho Nacional para o Desenvolvimento Científico e Tecnológico (CNPq), and Coordenação de Aperfeiçoamento de Pessoal de Nível Superior (CAPES).

### Conflict of interest statement

The authors declare that the research was conducted in the absence of any commercial or financial relationships that could be construed as a potential conflict of interest.
